# Use of a fixed, body weight-unadjusted loading dose of unfractionated heparin for extracorporeal cardiopulmonary resuscitation

**DOI:** 10.1186/s40560-015-0098-z

**Published:** 2015-07-21

**Authors:** Yoshiaki Iwashita, Mashiro Yukimitsu, Masaki Matsuduki, Akitaka Yamamoto, Ken Ishikura, Hiroshi Imai

**Affiliations:** Emergency and Critical Care Center, Mie University Hospital, Edobashi 2-174, Tsu, Mie Japan

**Keywords:** Heparin, Extracorporeal cardiopulmonary resuscitation, Activated coagulation time

## Abstract

**Background:**

Extracorporeal cardiopulmonary resuscitation (ECPR) is being used increasingly in the emergency and critical care field in Japan. A major complication of ECPR is bleeding; however, the optimal initial heparin dose and activated coagulation time (ACT) remain unknown. The aim of this study was to assess the appropriateness of our initial anticoagulation protocol.

**Methods:**

We retrospectively evaluated the initial heparin dose, ACT value, and incidence of bleeding and thrombotic complications in post-cardiopulmonary arrest patients who received a fixed, body weight-unadjusted loading dose of unfractionated heparin (3000 U) prior to veno-arterial extracorporeal membrane oxygenator (ECMO) between February 2011 and November 2013 at Mie University Hospital, Japan.

**Results:**

ACT was evaluated within 3 h of initiation of 32 consecutive ECPR patients. The mean heparin dose per body weight was 53.6 U/kg and the mean ACT was 231.3 s. In 17 patients, ACT exceeded 200 s. Three patients experienced fatal bleeding in the chest wall within 24 h of receiving ECMO. The mean heparin dose per kilogram body weight, mean initial ACT, and mean duration of cardiopulmonary resuscitation (CPR) did not statistically differ between the patients who experienced fatal bleeding and those who did not.

**Conclusions:**

Fixed-dose heparin of 3000-U bolus resulted in a mean heparin dose per kilogram body weight of 53.6 U/kg and an ACT of 231.3 s and experienced 3 out of 32 fatal bleedings. Further researches are warranted to optimize anticoagulation protocol for ECPR patients.

## Background

Veno-arterial extracorporeal membrane oxygenation (ECMO) is being used increasingly for extracorporeal cardiopulmonary resuscitation (ECPR) in Japan because it provides higher survival rates and better neurological outcomes [1, 2]. Despite the development of this new technique, bleeding remains a major complication associated with ECPR. In patients receiving ECMO, anticoagulants such as unfractionated heparin are frequently used. In patients starting respiratory ECMO, the Extracorporeal Life Support Organization (ELSO) recommends 50–100 U/kg of unfractionated heparin given as a bolus injection and a target activated coagulation time (ACT) of 1.5 times the normal value [3]. Ideally, the heparin dose should be carefully adjusted by referencing both the patient’s body weight and normal ACT value; however, in the emergency setting, it is extremely difficult to obtain these values. Therefore, for practical reasons, at our hospital, we decided to use a fixed, body weight-unadjusted loading dose of 3000 U of unfractionated heparin in all patients undergoing ECPR.

To our knowledge, no studies examining the optimal initial heparin loading dose and ACT for veno-arterial ECMO have been conducted. Therefore, we retrospectively assessed the initial heparin loading protocol for veno-arterial ECMO used at our hospital. To achieve this objective, we firstly analysed the relationship between heparin dose and ACT value in our hospital by comparing with ELSO’s recommendation. Secondly, we analysed the major bleeding complication in the study period.

## Methods

We retrospectively investigated patients who had experienced cardiopulmonary arrest and in whom veno-arterial ECMO was initiated at Mie University Hospital, Japan, between February 2011 and November 2013. The study protocol was approved by the institutional review board of Mie University Hospital.

A CAPIOX (Terumo, Tokyo, Japan) ECMO system with a heparin-coated pump and cannulae was used in all of the study patients. A 12- to 18-Fr arterial cannula and a 20-Fr venous cannula were used in adult patients. Twelve- or fourteen-Fr cannulae were used in paediatric patients (<15 years old).

Upon cardiopulmonary arrest, conventional cardiopulmonary resuscitation (CPR) was initiated in all of the study patients in accordance with the *American Heart Association Guidelines for CPR and ECC* (2010) without the use of any adjunct devices in either the pre-hospital or hospital setting [4, 5]. Indications for introducing ECMO were age less than 75 years and cardiac arrest time less than 60 min, and ECMO was initiated when requested by one of our Board of Emergency Medicine-certified physicians. Percutaneous cannulation was performed in the emergency room by using the Seldinger technique.

All the patients underwent hypothermia therapy (target body temp., 34 °C) for at least 24 h and were gradually rewarmed. Normal blood pressure was maintained by adjusting the flow of blood through the ECMO pump and by minimizing the use of inotrope and vasopressor agents. Intra-aortic balloon pumps were placed in patients with low pulse pressures. Continuous renal replacement therapy was initiated for all renal indications. Ventilator settings used were “lung rest”; positive end-expiratory pressure, 5–10 cm H_2_O; low respiratory rate (<12/min); and FiO_2_ <60 %.

A fixed, body weight-unadjusted loading dose of 3000 U of unfractionated heparin was administered to all the study patients, and unfractionated heparin was used for anticoagulation therapy during ECMO. ACT was evaluated by using a Hemochron microcoagulation system (Technidyne, New Albany, USA). ACT and body weight were determined upon admission to the intensive care unit. In three patients, body weight could not be determined due to the patient’s clinical status or death, so body weight was estimated from their age according to the Japanese Ministry of Health, Labour, and Welfare guidelines.

We recorded patient age, sex, initial heparin dose, and initial ACT value; incidence of bleeding or thrombotic complications occurring within 24 h of ECMO initiation; and survival and neurological outcomes after hospital discharge. Fatal bleeding was defined as any bleeding that required surgical or trans-arterial embolization to maintain hemodynamic stability; minor bleeding was defined as any bleeding that did not require surgical intervention. Thrombotic complications were assessed by visually checking for circuit clotting. Neurological outcomes were assessed by using the Cerebral Performance Category scale.

Continuous variables are presented as mean (range), and categorical variables are presented in the text as percentages. Continuous variables were compared between groups by using the Student’s *t* test. Categorical variables were compared by using the chi-square test. For all analyses, significance was defined as *P* < 0.05. All analyses were performed with statistical software (Statistical Package for the Social Sciences [SPSS] version 18; SPSS Japan Inc., Tokyo, Japan).

## Results

The anticoagulation protocol currently used in our hospital is shown in Table 1. The ECMO circuit is primed by using a fixed, body weight-unadjusted loading dose of 3000 U of heparin-infused normal saline. If cannulation takes longer than usual, an additional dose of heparin is administered. Two patients in the present study received a total of 7500 U of heparin, and one patient received a total of 5500 U of heparin. All other patients received 3000 U of heparin.

**Table 1:** Comparison of anticoagulation protocols

	Our protocol	ELSO guideline
Initial dose	3000 U^a^	50–100 U/kg
Additional dose	2000–5000 U	NA
Target ACT	180–200 s	1.5 times the normal value

*ACT* activated coagulation time, *ELSO* Extracorporeal Life Support Organization, *NA* not applicable

^a^Fixed and body weight unadjusted

**Fig 1:**
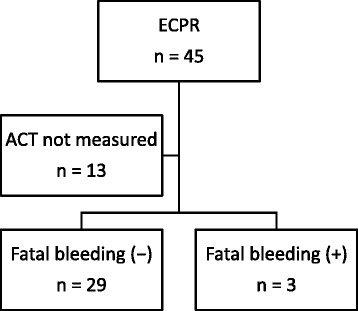
Study overview. Extracorporeal membrane oxygenation (ECMO) was initiated in 45 consecutive patients. Thirteen patients were excluded from the study because activated coagulation time (ACT) was not determined within 3 h of admission to the intensive care unit. The remaining 24 patients were included in the present study

ECMO was initiated in 45 patients during the study period (Fig. 1). In one patient, ECMO was initiated twice during the same hospitalization. In all other patients, ECMO was initiated once at each hospitalization. ACT was not measured within 3 h of admission to the intensive care unit in 13 patients so they were excluded from the study. Table 2 shows the patient demographics. The mean age was 60.4 years. Twenty-seven patients (84.3 %) were male. Nine patients (37.5 %) experienced cardiac arrest during hospitalization. The mean duration of conventional CPR prior to the initiation of ECMO was 48.6 min. The mean initial heparin dose was 53.6 U/kg body weight, and the mean initial ACT was 231.3 s.

**Table 2:** Patient demographics

Number of patients	32
Mean age	60.4 (12–87)
Sex (male to female ratio)	27:5
Mean initial heparin dose (U/kg)	53.6 (31.6–115.2)
Mean initial ACT (s)	231.3 (103–359)
Mean duration of conventional CPR (min)	48.6 (4–106)
Survival	9
CPC 1 or 2	6

**Fig 2:**
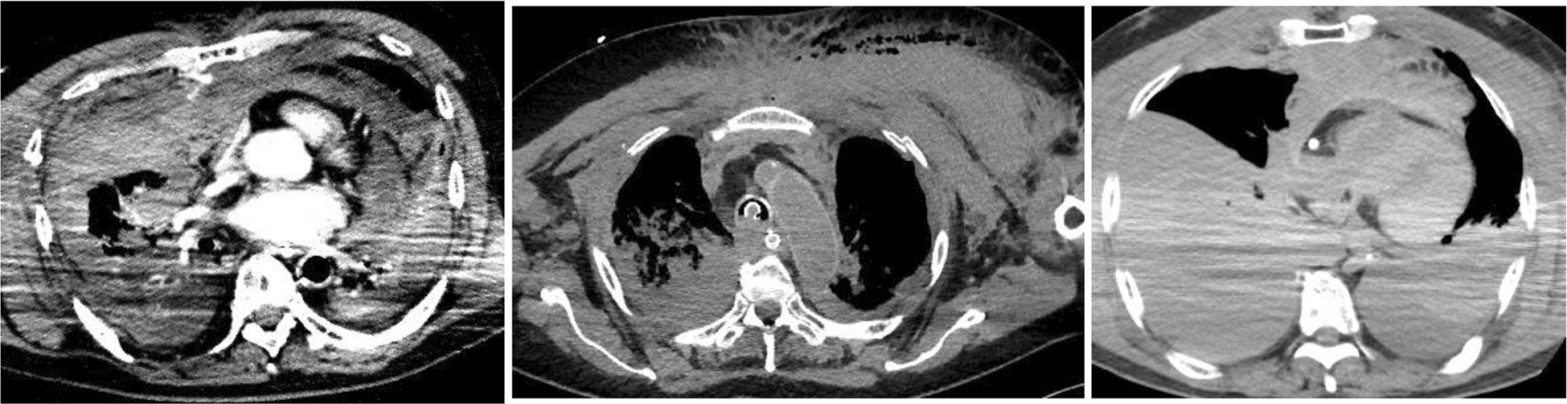
Computed tomography scans showing intra-thoracic, thoracic, and mediastinal bleeding, presumably caused by excessive chest compression, in the three patients in the present study who experienced fatal bleeding

Nine out of the thirty-two patients (28.1 %) survived to discharge, six of whom had a good neurological outcome (Cerebral Performance Category 1 or 2). The causes of death in the patients that died included cerebral hypoxia, low cardiac output syndrome, and multiple organ failure. Three patients experienced fatal bleeding during ECMO (Table 3), two of whom died as a consequence. The remaining patient underwent interventional radiology to stop the haemorrhage but died due to low cardiac output syndrome. Figure 2 shows computed tomography scans of the three patients who experienced fatal bleeding; thoracic, intra-thoracic, and mediastinal bleeding can be seen. There were no significant differences in age (*P* = 0.33), sex (*P* = 0.84), mean initial heparin dose (*P* = 0.42), mean initial ACT (*P* = 0.89), or mean duration of conventional CPR (*P* = 0.18) between the patients who experienced fatal bleeding and those who did not (Table 3). No significant thrombotic complications were observed.

**Table 3:** Comparison of cases with or without fatal bleeding

	Fatal bleeding (−)	Fatal bleeding (+)	*P* value
	(*n* = 29)	(*n* = 3)	
Age (mean)	59.5	69.3	0.33
Sex (male)	25	2	
Mean initial heparin dose (U/kg)	54.6	44.7	0.42
Mean initial ACT (s)	263.5	224.3	0.89
Mean duration of conventional CPR (min)	51.9	67	0.18

*ACT* activated coagulation time, *CPR* cardiopulmonary resuscitation

## Discussion

It is difficult to measure body weight prior to initiating ECMO in the emergency setting, so at our hospital, we decided to use a fixed, body weight-unadjusted loading dose of heparin as anticoagulation therapy. Therefore, all the patients received 3000 U of heparin; three patients received an additional dose of heparin. As a result, the initial mean heparin dose per kilogram body weight was 53.6 U/kg and ACT of 231.3 s, and experienced 3 out of 32 fatal bleedings. Although the ELSO guideline recommends a heparin bolus injection of 50–100 U/kg, there is no definitive evidence showing that this dose is sufficient to minimize mortality or morbidity [3]. Indeed, a recently published multicentre prospective trial did not use a fixed anticoagulation protocol for starting ECPR [1]. Furthermore, the ELSO guideline was originally intended for respiratory ECMO, so the appropriate dose of initial heparin for ECPR patients remains unknown.

According to the ELSO database, the currently recommended ACT value, 150–180 s, resulted in a rate of bleeding complications of 30–35 %; however, these data were obtained in the respiratory ECMO patients only and more than 50 % of these patients were veno-venously cannulated [6]. Patients who undertake percutaneous coronary intervention (PCI) are recommended to receive 70–100 U/kg of unfractionated heparin and resultant ACT of >250 s before PCI [7]. One of the reasons that ECMO patients require a lower dose of heparin with a shorter ACT value may be due to patient’ disease state. Indeed, in PCI patients, the severity of disease state is correlated with the incidence of post PCI bleeding [8]. Patients who need ECMO are usually critically ill with deteriorated coagulation status. Therefore, a study specifically examining anticoagulation strategy for ECPR patients is needed.

Postcardiotomy patients receiving veno-arterial ECMO frequently experience bleeding complications. Rastan et al. analysed 517 consecutive postcardiotomy cardiogenic-shock patients who required extracorporeal circulation and found that 300/517 (58.0 %) patients underwent rethoracotomy for bleeding complications [9]. Furthermore, Doll et al. reported that 136/219 (63.5 %) postcardiotomy patients who had undergone ECMO experienced severe mediastinal bleeding requiring rethoracotomy [10]. These data suggest that the underlying etiology and type of cannulation may influence the rate of bleeding complications.

Since post-cardiac arrest patients have better neurological outcomes when treated with induced hypothermia [11, 12], induced hypothermia therapy is often initiated together with ECPR. In the present study, all the patients underwent mild hypothermia therapy (target temp., 34 °C). Hypothermia is associated with increased risk of bleeding [13, 14]. Chest compression is also associated with bleeding complications. The current recommendation for chest compression in patients in cardiac arrest is to push more than 3 cm; however, chest compression exceeding 6 cm is associated with a greater incidence of complications [15]. Therefore, vigorous chest compression should be avoided in patients in whom ECPR will be introduced. Furthermore, compared with patients in acute respiratory failure receiving respiratory ECMO, patients receiving ECPR, as well as postcardiotomy patients, have a higher risk of bleeding. An improved anticoagulation protocol is needed for these high-risk patients.

There were three cases of fatal bleeding related to chest compression among the patients examined in the present study. The mean initial heparin dose, mean initial ACT value, and mean duration of conventional CPR were not statistically associated with the incidence of fatal bleeding during ECMO (Table 3). Details of the factors contributing to these thoracic bleedings cannot be detected with this small-sized observational study; however, rib fractures can clearly be seen near the hematoma sites on computed tomography scans, so it is likely that chest compression was a contributing factor (Fig. 2). However, postmortem CT study for conventional CPR patients has shown that the incidence of rib fracture was 70 %, while hemothorax was seen in only 0.45 % in the conventional CPR patients [16]. Since our patient group experienced 3 out of 32 (9.3 %) fatal chest hematoma, we assume that chest hematoma is exacerbated by our anticoagulation therapy for ECPR.

There are several reports indicating the safety of using a heparin-free ECMO circuit. Muehrcke et al. reported the details of 30 individual cases of heparin-free ECMO use; bleeding complications were reported in 12/30 (40 %) patients and 6 cases of intracardiac clotting were reported [17]. In a prospective analysis of 32 postcardiotomy patients receiving veno-arterial ECMO, Lamarche et al. reported that 14 (43.8 %) patients required rethoracotomy and that 2 patients experienced intracardiac thrombus [18]. Recently, there are several reports indicating IABP patients can be managed safely without heparin when used for postcardiotomy patients [19, 20]. Together, these results suggest that the use of heparin-free circuits may lower the overall incidence of bleeding complications in postcardiotomy patients receiving ECMO.

Few studies have examined clotting events. However, Rastan et al. examined the findings of 78 autopsies of patients who had received ECMO. They reported thromboembolic findings in a total of 46.2 % cases, 15.4 % of which were known prior to death, indicating that clinically visible thromboembolic events occur much less frequently than subclinical thromboembolic events do [21]. In the present study, we did not examine thrombotic complications in detail; circuit clotting was only checked for by means of visual observation. It is, however, important to carefully monitor patients receiving ECMO for potential subclinical thromboembolic events.

The use of the anticoagulant nafamostat as an alternative to heparin is currently being investigated. Han et al. described the usefulness of nafamostat for both cardiac and respiratory ECMO; patients receiving nafamostat received less red blood cells and fresh frozen plasma compared with patients receiving heparin [22]. The incidence of haemorrhage or thrombosis was also lower in the nafamostat group. Further studies are needed to clarify how to avoid fatal bleeding in patients receiving ECPR.

## Conclusions

Fixed-dose heparin of 3000-U bolus resulted in a mean heparin dose per kilogram body weight of 53.6 U/kg and an ACT of 231.3 s, and experienced 3 out of 32 fatal bleedings. Further research is warranted to optimize appropriate anticoagulation protocol for ECPR patients. At this point, avoiding vigorous chest compression and reducing anticoagulating agents are the possible intervention to minimize bleeding complications safely.
